# Computational modelling and analysis of the molecular network regulating sporulation initiation in *Bacillus subtilis*

**DOI:** 10.1186/s12918-014-0119-x

**Published:** 2014-10-24

**Authors:** Adaoha EC Ihekwaba, Ivan Mura, Gary C Barker

**Affiliations:** Gut Health and Food Safety, Institute of Food Research, Norwich Research Park, Colney, Norwich, UK; Faculty of Engineering, EAN University, Carrera 11 No. 78 – 47, Bogotá, Colombia

**Keywords:** Systems biology, Computational modelling, Sensitivity analysis, Signal transduction, Sporulation, *Bacillus subtilis*

## Abstract

**Background:**

Bacterial spores are important contaminants in food, and the spore forming bacteria are often implicated in food safety and food quality considerations. Spore formation is a complex developmental process involving the expression of more than 500 genes over the course of 6 to 8 hrs. The process culminates in the formation of resting cells capable of resisting environmental extremes and remaining dormant for long periods of time, germinating when conditions promote further vegetative growth. Experimental observations of sporulation and germination are problematic and time consuming so that reliable models are an invaluable asset in terms of prediction and risk assessment. In this report we develop a model which assists in the interpretation of sporulation dynamics.

**Results:**

This paper defines and analyses a mathematical model for the network regulating *Bacillus subtilis* sporulation initiation, from sensing of sporulation signals down to the activation of the early genes under control of the master regulator Spo0A. Our model summarises and extends other published modelling studies, by allowing the user to execute sporulation initiation in a scenario where Isopropyl β-D-1-thiogalactopyranoside (IPTG) is used as an artificial sporulation initiator as well as in modelling the induction of sporulation in wild-type cells. The analysis of the model results and the comparison with experimental data indicate that the model is good at predicting inducible responses to sporulation signals. However, the model is unable to reproduce experimentally observed accumulation of phosphorelay sporulation proteins in wild type *B. subtilis*. This model also highlights that the phosphorelay sub-component, which relays the signals detected by the sensor kinases to the master regulator Spo0A, is crucial in determining the response dynamics of the system.

**Conclusion:**

We show that there is a complex connectivity between the phosphorelay features and the master regulatory Spo0A. Additional we discovered that the experimentally observed regulation of the phosphotransferase Spo0B for wild-type *B. subtilis* may be playing an important role in the network which suggests that modelling of sporulation initiation may require additional experimental support.

**Electronic supplementary material:**

The online version of this article (doi:10.1186/s12918-014-0119-x) contains supplementary material, which is available to authorized users.

## Background

Spore-forming bacteria are a major cause of food spoilage and disease, and food industries actively employ strategies to ensure adequate inactivation of spores and the control of outgrowth, both for species that potentially lead to spoilage and for foodborne pathogens. The contamination of foods with bacterial spores is well documented in many scenarios [1,2]. Spore formers need specific conditions for germination and growth, which can occur in food, such as low or high temperatures and anaerobic or acidophilic conditions [3,4], sometimes in combination.

Most microbial spore forming bacteria respond to stress (e.g., nutrient deprivation) by inducing the expression of an appropriate suit of adaptive (stress-response) genes to help them cope with adverse environmental circumstances; an extreme example is endospore formation. The initiation of sporulation is one of the decisive moments in the life cycle of sporulating bacteria, whereby an extremely durable cell called a spore is formed, as exemplified by the bacterium *Bacillus subtilis*.

The decision to abandon vegetative growth and enter sporulation has far reaching consequences for *B. subtilis*. It involves a switch between two completely different genetic programs requiring energy-expensive changes in gene expression and cell morphology [5,6]. These changes are regulated by a complex network involving more than 120 genes [5,7]. The size and complexity of the sporulation network make it difficult to interpret the switch from vegetative growth to sporulation in terms of the interactions between genes, proteins, and small molecules. This is where computer modelling and simulation is able to help. Mathematical modelling is utilized in understanding how a global response can emerge from a network of local interactions [8-10]. In addition, the use of modelling and simulation tools may permit further, the formulation of hypotheses on missing components and interactions which, after a process of reiterative computer simulation, can guide future experimentation.

Various mathematical models of *B. subtilis* sporulation mechanisms can be found in the literature [6,11-19], however, for the purpose of our study we only mention a small list of them here. We start with the work of Narula and colleagues, who in [16] defined a computational model which was used to generate a molecular explanation of how the cell integrates and relays its environmental signals to the master regulator of the sporulation initiation process, Spo0A. They employed an inducible promoter system whereby the activation of KinA, the upstream phosphoryl donor in the sporulation network, was regulated through the addition of IPTG to the medium. Moreover, in their more recent study [18], they further considered a doubly inducible system whereby another kinase, KinC, was expressed from an inducible IPTG promoter, while Spo0A was expressed from a xylose-inducible promoter. Based on the experimental analysis of these systems, they were able to define a mechanistic model supporting a conjecture on how important the proper activation pattern of Spo0A was to the system and how its early accumulation may impair the ability of the cell to complete sporulation. Other groups, Sen and colleagues, as well as Kothamachu and colleagues performed also modelling studies, however, their main focus was on the four component *B. subtilis* phosphorelay, the key module in the molecular network that transduces the environmental signals captured by the sensing kinases towards the master regulator Spo0A [14,17]. While Sen and colleagues [17] considered the complex positive and negative feedback loops thought to exist between Spo0A and the upstream proteins of the phosphorelay; as well as how the modulation induced by *B. subtilis* cell cycle on the synthesis of phosphorelay proteins affects the dynamics of Spo0A; Kothamachu *et. al*., in their work [14] modelled the interactions between the four components of the phosphorelay to study the dependence of the Spo0A steady state activation level on the kinetic parameters of the phospho-transfer reactions – without taking into consideration the feedback loops among species. Another group, Kuchina and colleagues [15], took a different perspective in their modelling, by trying to solve the problem of whether the molecular machinery regulating the sporulation process in *B. subtilis* proceeds through a sequence of reversible or irreversible decision-making steps. To do this, the authors employed a different modelling approach, whereby the impact of fluctuating environmental conditions was modelled with the aim of identifying the exact point at which cells become committed to sporulation – abstracting the molecular details of the sporulation network.

Since it is known that *B subtilis* exhibits ultra-sensitivity to sporulation stimuli [16], characterizing the step at which sporulation decision is made could provide hints on the upstream molecular dynamics. The experimental data provided by [16] has shown that the best matching scenario is one when the initial progression towards sporulation commitment is reversible (with an irreversible later stage), with commitment arising downstream of Spo0A activation. This finding would therefore suggest that the switch mechanism leading to the ultra-sensitivity is to be placed downstream of Spo0A activation, in the area of the sporulation network which should lead to the sustained expression of the sigma factors that control later sporulation genes.

After reviewing the aforementioned works, the purpose of our study was to understand how the current state-of-the-art in the modelling of *B. subtilis* sporulation – which so far has been based on the use of inducible systems – can be used to explain the behaviour of the organism, including wild type strains. To this end, we first defined a sporulation model that borrowed generously information from the above-mentioned studies and summarised the current knowledge of the *B. subtilis* sporulation network at the molecular level. The scope of our model is limited to the chain of molecular interactions that are triggered by the inducers, which are then forwarded through the phosphorelay, ultimately leading to the activation of the master regulator Spo0A, and to the expression of the *spoIIA*, *spoIIE* and *spoIIG* genes. This model, which is implemented in the modelling and simulation package COPASI, is available for download in the COPASI native format (Additional file 1) and in SBML as well (Additional file 2), to facilitate checks of all the results presented in this paper. We analyzed the model output and performed a validation against published experimental data. We use transient, steady state and parameter sensitivity analysis to determine, among model components and parameters, the most important quantities in determining the predicted behaviour.

The results of our analysis suggest that the response to artificial sporulation signals in inducible systems is quantitatively and qualitatively different from the one observed in wild type and cannot be predicted with models of current biological knowledge. Specifically, in wild type a slower and almost linear pattern of Spo0A activation is observed, in contrast with the switch-like behaviour that is observed for the inducible models. This conclusion is supported by a number of remarks that can be found dispersed in the literature, which point to the need for a gradual accumulation of active Spo0A (see for instance [18] and [20]) or the influence that the cell-cycle has on slowing-down Spo0A accumulation (see [17] and [19]). Moreover, our analysis highlights components and interactions in the phosphorelay that require a further characterization. Indeed, some experimental evidence of regulated synthesis for the phosphorelay component Spo0B was not included in the published models, and the reaction kinetics used in our model, which are largely extracted from the literature, may not provide a faithful representation of the molecular mechanism for the wild type behaviour.

## Methods

In this section, we provide a detailed description of the mathematical model as well as the methods used for analysis.

### *B. subtilis* sporulation network

The DNA-binding protein Spo0A is the master regulator for entry into sporulation. The capacity of the protein to alter transcription is governed both by accumulation of the protein and its state of phosphorylation. Phosphorylation of Spo0A causes the re-orientation of a phenylalanine residue in the molecule, allowing an effector domain to become active [21].

Spo0A phosphorylation is controlled by a multicomponent phosphorelay capable of integrating diverse physiological and environmental signals. The *B. subtilis* phosphorelay is comprised of at least five sensor histidine kinases – termed KinA, KinB, KinC, KinD, and KinE - a secondary messenger, Spo0F, a phosphotransferase, Spo0B, and the response regulator Spo0A. The presence of multiple phosphorelay components in this organism is thought to provide more points of regulation and increased precision for the response as each step reinforces discrimination [22]. Spo0A is both an activator and a repressor of gene transcription. As an activator, Spo0A acts in conjunction with RNA polymerase containing either the housekeeping sigma factors sigma A or the alternative sigma factor, sigmaH, by binding to cognate sequences in the promoter regions of genes under its control [23]. Binding of Spo0A~P to DNA directly affects the expression of approximately 121 genes [24]. Among these are key genes that drive the positive regulation of sporulation - particularly the *spoIIA* [25,26], *spoIIE* [27] and *spoIIG* [28,29] genes involved in establishing compartment-specific transcription under the control of σ^F^ (*spoIIA* operon and the *spoIIE* gene) and σ^E^ (*spoIIG* operon) [30]. Acting as a repressor, Spo0A blocks expression of the *abr*B gene, a gene which encodes the “transition state” regulator, AbrB, which is itself a repressor of sigmaH [24,31]. Spo0A mediated repression of *abr*B leads to the depletion of the AbrB protein from the cell and to the accumulation of sigmaH, which results in the activation of genes under its positive control - which includes KinA, Spo0F and the Spo0A gene itself [32,33]. Therefore, Spo0A-mediated repression of the *abr*B sets up a self-reinforcing cycle that contributes to the accumulation of Spo0A protein at the start of the sporulation [24,33].

#### Brief summary of the model

**Figure 1:**
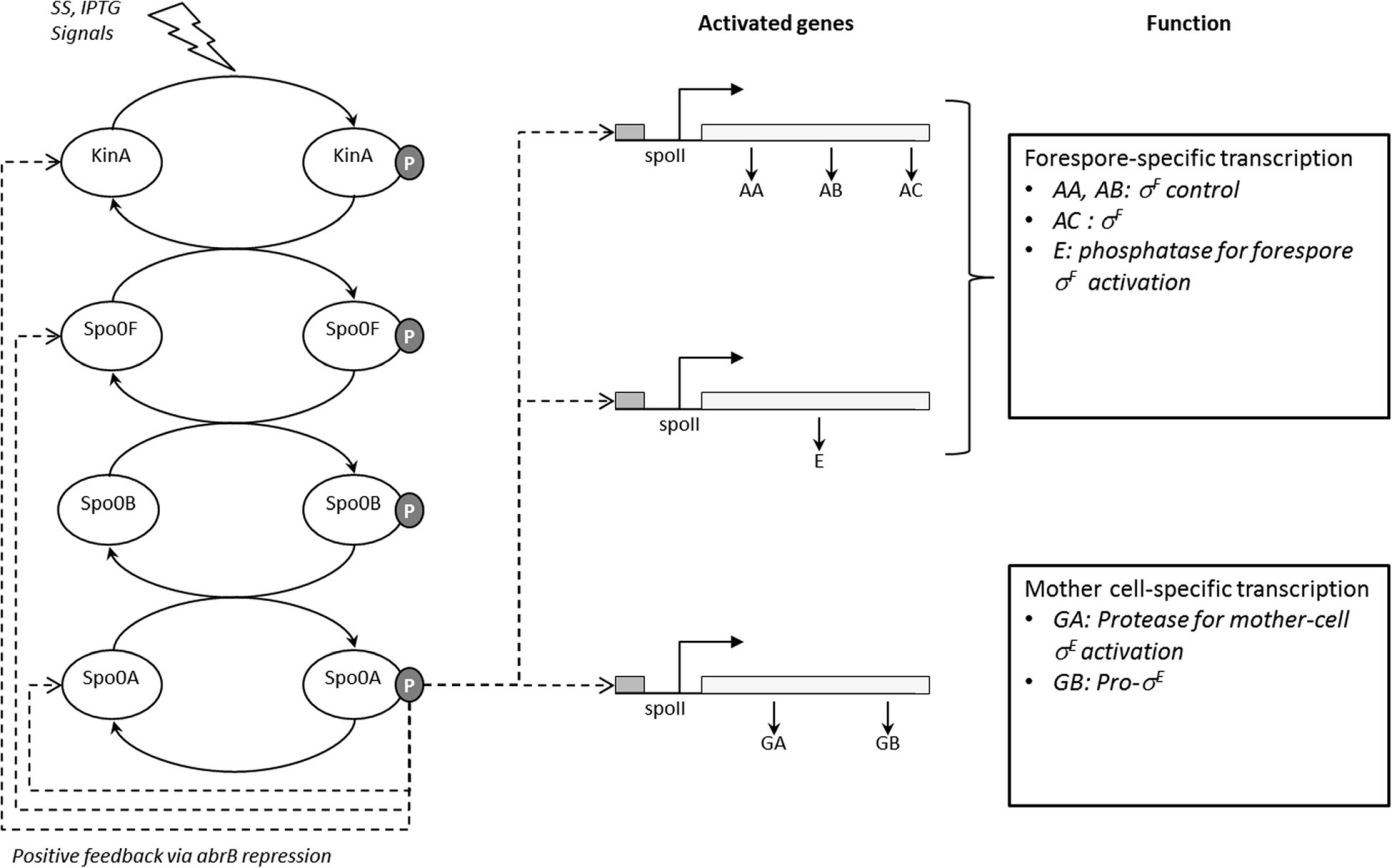
**The sporulation initiation network in** ***B. subtilis*** **.** The histidine kinase KinA responds to the environmental conditions (SS) and to artificial induction (IPTG) by auto-phosphorylation and pass phosphoryl groups to an intermediate response regulator, Spo0F. Subsequently, the phosphotransferase Spo0B, transfers it to the transcription factor, Spo0A. Spo0A~P activates the genes that govern forespore– and mother cell–specific transcription factors and exerts a positive feedback on phosphorelay components through the repression of *abr*B gene expression.

The architecture of the molecular network model governing sporulation initiation in *B. subtilis* can be described diagrammatically as in Figure 1.

Out of the five kinases identified as capable of initiating sporulation in *B. subtilis* [34], we have considered KinA, the major kinase responsible for initiation of sporulation in our model. KinA overexpression during exponential growth is sufficient to induce entry into sporulation [20]. Observations on how specific sporulation-inducing signals affect the activation of this protein are available in literature [35,36].

We consider two distinct types of induction signals that drive the cell into sporulation. One is a sporulation signal (SS, in the model), which we use to represent the influence of the adverse environment on the auto-phosphorylation of KinA (the molecular details of SS are still unknown). The other signal is the commonly used artificial sporulation initiation system where KinA expression level is controlled from an isopropyl-β-D-thiogalactopyranoside (IPTG)–inducible promoter. IPTG is a popular chemical inducer that can be used to exogenously control the transcriptional activity of wild-type or synthetic lactose repressor (LacI) in order to manipulate the production of KinA proteins in bacteria [37]. Use of IPTG allows for tuneable control of KinA expression [38,39]. Hence, we can feed our model with different stimuli that together can represent either an artificial induction, independent of the environmental conditions, or a natural induction occurring in wild type.

The sporulation signal we introduce is modelled as an abstract species SS, which controls the autophosphorylation of KinA dimers [40-42]. The IPTG regulation effect on the tuning of the KinA levels has been modelled by following some of the assumptions available in literature [16,38,43,44]. We model IPTG regulation of sporulation initiation through the indirect release of KinA synthesis inhibition by LacI, which when successfully incorporated into a promoter, has been used to regulate the expression of many genes. LacI is a DNA-binding transcription factor that represses transcription of the operon involved in transport and catabolism of lactose [43,45]. This binding is altered by the presence of sugar ligands (inducers), which elicit a conformational change in the protein to a state with lower affinity for the operator DNA sequence. When lactose becomes available, it is converted into allolactose, binds to the lac repressor, causing an allosteric change in its shape (LacI_d). In its changed state, the lac repressor is unable to bind tightly to its cognate operator. This effect is referred to as induction, because it induces, rather than represses expression of the metabolic genes. In vitro, IPTG is commonly used as an allolactose mimic to induce transcription of genes being regulated by lac repressor. IPTG binds to Lac-repressor (LacI) and deactivates it by bringing it to the inactive form LacI_d, this way reducing the effective free Lac-repressor concentration that would negatively affect KinA levels. Therefore, in the presence of IPTG, expressed KinA levels can be increased.

In our model, KinA is the phosphoryl donor that activates the *B. subtilis* phosphorelay. We include the main phosphorelay species Spo0F, Spo0B and Spo0A [46], abstracting the effects of the transition-state regulators Sin I/R (sporulation inhibitor (SinR) and its antagonist (SinI) [47-49]), based on the results reported by Chastanet *et al.* [12]. Chastanet and colleagues reported in [12] that these regulators were quickly repressed early on in sporulation and thus have little effect on the dynamics of Spo0A. Similarly, we do not explicitly consider the *abrB* repression induced by Spo0A~P, but we model the impact of AbrB depletion on the phosphorelay species KinA, Spo0F and Spo0A, by a Spo0A~P induced positive feedback [32].

Dephosphorylation of Spo0A has been shown to be carefully monitored by cells at all times during the cell cycle through two families of aspartyl-phosphate phosphatases – the Rap and Spo0E families [50,51], which provide opportunity for negative signals to influence the cell’s decision whether to sporulate or continue with vegetative growth. RapA, B and E indirectly inhibit the phosphorylation of Spo0A via dephosphorylation of Spo0F [34,50,52,53], whilst Spo0E dephosphorylates Spo0A directly [51,54,55]. To simplify our modelling, Rap and Spo0E phosphatases are only taken into consideration in an abstract manner. The dephosphorylation of Spo0F and Spo0A proteins are modelled as first order reactions assuming that Rap and Spo0E concentrations are not rate limiting.

Spo0A~P up-regulates transcription from *spoIIA*, *spoIIE*, and *spoIIG* promoters [26-29]. *spollA_t* mRNAs are translated into AA, AB and AC proteins, *spollE_t* mRNAs are translated into IIE protein molecules and *spollG_t* mRNAs are translated into GA and GB protein molecules. Following the modelling approach proposed by Narula *et. al.* in [16], we model the dynamics of transcription up-regulation as being the result of a cooperative binding of active Spo0A to the promoters. Moreover, for each species, apart from SS and IPTG, we model the synthesis and degradation processes. Transcription is always modelled as a single chemical reaction synthesizing mRNA where the synthesis rate is controlled by promoter strengths. Translation is modelled as a single chemical reaction with a rule proportional to the amount of corresponding mRNA.

#### Design and implementation

**Figure 2:**
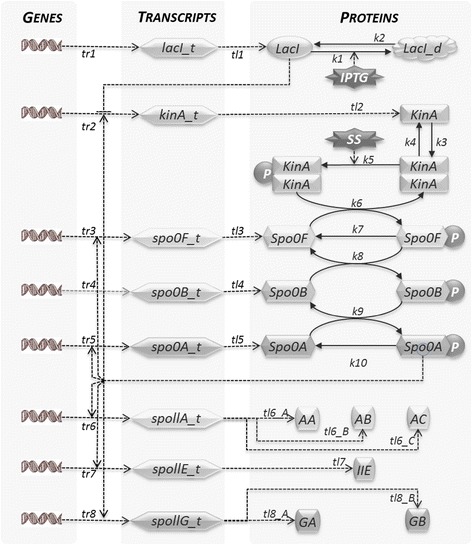
***B. subtilis*** **sporulation network model architecture.** This cartoon model represents the sporulation initiation in *B. subtilis*. IPTG relieves LacI repression on the gene coding for KinA. KinA dimer auto-phosphorylation is regulated by an unknown sporulation signal (SS), which is generated by the unfavourable environmental conditions (lack of nutrients or high density population). Activated KinA transfers phosphor groups to Spo0F, which then leads to Spo0A phosphorylation via the phosphotransferase Spo0B. Dephosphorylation of Spo0F and Spo0A is modelled by abstracting the phosphatase species Spo0E and RapA. Spo0A~P acts as a transcription factor, and activates gene transcription by binding to the spoll promoters. mRNAs are translated into important sporulation initiation proteins, AA, AB, AC, IIE, GA and GB.

Figure 2 shows a cartoon of the model system. For the sake of clarity, the degradation reactions of mRNAs and protein molecules are not included in the graphical representation of the model.

The graphical notation used in the diagrammatic representation identifies species as shapes and reactions as arrows. A direct arrow from species A to species B indicates a reaction where A is a reactant and B is a product. Solid arrows stand for mass transfer reactions, i.e. reactions that consume reactants to produce products, whereas dashed arrows indicate reactions where the abundance of a species regulates a reaction rate, but the reactant concentration is not affected by the occurrence of the reaction. For every reaction, we define in the model a rate function, whose exact mathematical form is detailed in Table 1.

**Table 1:** **Parameter values used for the modelling**

**Rate**	**Description**	**Mathematical form**	**Parameter values**	
*tr1*	LacI transcription	*constant: k*	*k = 0.1 nMs* ^*−1*^	*Fitted*
*tl1*	LacI translation	*mass action: k · [lacI_t]*	*k = 0.2 s* ^*−1*^	*Fitted*
*k1*	LacI inactivation by IPTG	*mass action: k · [lacI_t] · [IPTG]*	*k = 0.000175 nM* ^*−1*^ *s* ^*−1*^	[44]
*k2*	LacI reactivation	*mass action: k · [LacI_d]*	*k = 0.016 s* ^*−1*^	[44]
*tr2*	KinA transcription	*hill function:* $\left({k}_1+{k}_2\frac{K{L}^2}{K{L}^2+{\left[ Lac\_I\right]}^2}\right)\left(1+{k}_3\frac{{\left[Spo0A\sim P\right]}^2}{K{S}^2+{\left[Spo0A\sim P\right]}^2}\right)$	*k1 = 0.0275 nMs* ^*−1*^	[16,24]
			*k2 = 0.24 nMs* ^*−1*^	
			*k3 = 1.95 nMs* ^*−1*^	
			*KL = 50 nM*	
			*KS = 2100 nM*	
*tl2*	KinA translation	*mass action: k · [kinA_t]*	*k = 0.0659 s* ^*−1*^	*Fitted*
*k3*	KinA dimerization	*mass action: k · [KinA]* ^*2*^	*k = 0.001 nM* ^*−1*^ *s* ^*−1*^	*Fitted*
*k4*	KinA dimer dissociation	*mass action: k · [KinA:KinA]*	*k = 0.25 s* ^*−1*^	*Fitted*
*k5*	KinA dimer auto-phosphorylation	*mass action: k · [KinA:KinA] · [SS]*	*k = 0.001 nM* ^*−1*^ *s* ^*−1*^	*Fitted*
*tr3*	Spo0F transcription	*hill function:* ${k}_1+{k}_2\frac{{\left[Spo0A\sim P\right]}^2}{K{S}^2+{\left[Spo0A\sim P\right]}^2}$	*k1 = 0.02 nMs-1*	[16,24]
			*k2 = 0.1 nMs-1*	
			*KS = 50 nM*	
*tl3*	Spo0F translation	*mass action: k · [Spo0F]*	*k = 0.0723 s-1*	[16]
*k6*	Spo0F phosphorylation	*mass action: k · [Spo0F] [KinA:KinA~P]*	*k = 0.00039 nM* ^*−1*^ *s* ^*−1*^	[64]
*k7*	Spo0F dephosphorylation	*mass action: k · [Spo0F~P]*	*k = 0.05 s* ^*−1*^	
*tr4*	Spo0B transcription	*constant: k*	*k = 0.2384 nMs* ^*−1*^	*Fitted*
*tl4*	Spo0B translation	*mass action: k · [Spo0B]*	*k = 0.1076 s* ^*−1*^	*Fitted*
*k8*	Spo0B phosphorylation	*mass action: k · [Spo0B] [Spo0F~P]*	*k = 0.00001 nM* ^*−1*^ *s* ^*−1*^	[64]
*tr5*	Spo0A transcription	*hill function:* $k1\frac{Kk1}{Kk1+\left[Spo0A\sim P\right]}+k2\frac{{\left[Spo0A\sim P\right]}^2}{Kk{2}^2+{\left[Spo0A\sim P\right]}^2}$	*k1 = 0.01388 nMs* ^*−1*^	[16]
			*k = 0.1388 nMs* ^*−1*^,	
			*Kk1 = 100 nM,*	
			*Kk2 = 150 nM*	
*tl5*	Spo0A translation	*mass action: k · [Spo0A]*	*k = 0.2143 s* ^*−1*^	*Fitted*
*k9*	Spo0A phosphorylation	*mass action: k · [Spo0A] [Spo0B~P]*	*k = 0.0008 nM* ^*−1*^ *s* ^*−1*^	[64]
*k10*	Spo0A dephosphorylation	*mass action: k · [Spo0~P]*	*k = 0.05 s* ^*−1*^	
*tr6*	spollA transcription	*hill function:* $\mathrm{k}1+\mathrm{k}2\frac{{\left[\mathrm{S}\mathrm{p}\mathrm{o}0\mathrm{A}\sim \mathrm{P}\right]}^4}{{\mathrm{Kk}}^4+{\left[\mathrm{S}\mathrm{p}\mathrm{o}0\mathrm{A}\sim \mathrm{P}\right]}^4}$	*k1 = 0.0277 nMs* ^*−1*^	[16,24]
			*k2 = 0.4166 nMs* ^*−1*^	
			*Kk = 140nM*	
*tl6_A*	AA translation	*mass action: k [spollA_t]*	*k = 0.1250 s* ^*−1*^	*Fitted*
*tl6_B*	AB translation	*mass action: k [spollA_t]*	*k = 0.0555 s* ^*−1*^	*Fitted*
*tl6_C*	AC translation	*mass action: k [spollA_t]*	*k = 0.0138 s* ^*−1*^	*Fitted*
*tr7*	spollE transcription	*hill function:* $k1+k2\frac{{\left[Spo0A\sim P\right]}^4}{K{k}^4+{\left[Spo0A\sim P\right]}^4}$	*k1 = 0.0208 nMs* ^*−1*^	[16,24]
			*k2 = 0.3125 nMs* ^*−1*^	
			*Kk = 230 nM*	
*tl7*	IIE translation	*mass action: k [spollE_t]*	*k = 0.0138 s* ^*−1*^	[16]
*tr8*	spollG transcription	*hill function:* $k1+k2\frac{{\left[Spo0A\sim P\right]}^4}{K{k}^4+{\left[Spo0A\sim P\right]}^4}$	*k1 = 0.0222 nMs* ^*−1*^	[16,24]
			*k2 = 0.7290 nMs* ^*−1*^	
			*Kk = 1700 nM*	
*tl8_A*	GA translation	*mass action: k [spollG_t]*	*k = 0.0034 s* ^*−1*^	[16]
*tl8_B*	GB translation	*mass action: k [spollG_t]*	*k = 0.0138 s* ^*−1*^	[16]

As shown in Figure 2, the LacI gene is constitutively expressed. The LacI repression of *kinA* gene expression is encoded as a negative cooperative effect, whereby the *kinA_t* mRNA is produced at a rate inversely proportional to *LacI*^*n*^, with *n = 2*. The deactivation effect of *IPTG* on *LacI* is modelled through the reversible sequestering of *LacI* into an inactive form *LacI_d* that is not capable of repressing the *kinA* gene expression [44]. The transcription of the *kinA* gene is also regulated by the feedback loop exerted by Spo0A~P. According to [39], we assume that KinA forms a homo-complex [42] via its N- and C-terminal domains and that KinA auto-phosphorylation is activated in response to an unspecified “sporulation signal” modelled by the SS species.

The phosphorelay circuit of our model includes the main phosphorelay species Spo0F, Spo0B and Spo0A, the phosphorylation, dephosphorylation and phosphotransfer reactions, and also synthesis and degradation of all species. Transcription of spo0F and spo0A genes is regulated by the amount of Spo0A~P. The dephosphorylation of Spo0F and Spo0A proteins are modelled as simple first order reactions, and due to the lack of knowledge about the regulation of phosphotransferase Spo0B, we do not include in the model any transcriptional control nor dephosphorylation reactions for this species. However, it is important to note that, according to experimental evidence [56], the concentration of Spo0B increases considerably upon sporulation initiation in wild type *B. subtilis*, suggesting the existence of additional regulation mechanisms. Finally, Spo0A ~ P up-regulates transcription from *spoIIA*, *spoIIE*, and *spoIIG* promoters [26-29]. Following the assumptions in Narula *et.al* [16], we have reflected these regulations in our model through cooperative activation effects on the transcription rates. *spollA_t* mRNAs are translated into AA, AB and AC proteins, *spollE_t* mRNAs are translated into IIE protein molecules and *spollG_t* mRNAs are translated into GA and GB protein molecules. As for the species and reactions considered here, this model is an extension of the model presented by [16] for IPTG inducible systems, in that details of the IPTG activity taken from [44], an additional stimulus (SS) representing the natural induction of sporulation in wild-type, and a positive feedback loop from Spo0A~P to KinA were all added to the model. Concerning the details of the kinetics of reactions, we obtained a number of the parameters from the literature. It is noteworthy that there is no consensus on some important parameters –e.g., the details of the kinetics of the regulated transcription of the phosphorelay species and of the downstream spoll targets, which are not exactly known and are inconsistently reported. We report in Table 1 the source of the specific kinetic information for each of the reactions we defined in our model.

### Model analysis methods

The overall model includes 13 species and taking into consideration transcripts, dimerization and post-translational modifications, therefore gives rise to 27 distinct forms. The total number of unidirectional reactions included in the model is 55. To analyse such a large model, we encoded the biochemical reactions in the COPASI software package [57], and used the algorithms implemented within the software for analysis.

The model was analysed using COPASI by adopting a continuous interpretation of the variables, with values that represent concentrations of molecular species, and a deterministic interpretation of the reaction rates, so that the reaction rates provided in the previous tables represent variation terms in the first time derivative of the concentrations. The model is encoded as a set of coupled ordinary differential equations. Therefore, the time-dependent transient behaviour of the model has been computed through numerical integration of the differential equations and the steady-state analysis by the solution of the set of polynomial equations that is obtained by setting to zero the value of the time derivatives in the differential equations.

In modelling and simulation, parameters are distinguished as being entities of a model that are either constant, under our direct control or vary independently - examples of which are kinetic constants and time. Variables are entities whose values are entirely determined by parameters - examples of which are the internal reactant concentrations and fluxes (and of course any other quantities calculated from these) [58]. Sensitivity analysis is an important tool used to study the dependence of a system on external parameters [9,59], and sensitivity considerations often play an important role in the design of control systems [60]. Parameter sensitivity analysis can also be utilized to validate a model’s response, and iteratively to design experiments that support the estimation of parameters [61].

We conducted parametric sensitivity analyses to determine how our model’s behaviour depends on its parameter values. Parametric sensitivity analysis can be divided into global sensitivity analysis (which addresses wide variations in parameter values), and local sensitivity analysis (which addresses small variations around a nominal operating condition). For our study, we exploited the local sensitivity analysis features of COPASI, which allows predicting the effect of small changes to the parameter on the model’s behaviour through the computation of relative sensitivity coefficients. The absolute values of coefficients provide insights into the model’s robustness, with small sensitivity coefficient indicating robustness with respect to perturbations of a parameter, and large coefficients suggesting high sensitivity. We also conducted global sensitivity analysis for selected parameters, following the indications provided by the local sensitivity results.

## Results

In this section we report and comment on the model outcomes, which we obtained by transient, steady-state and sensitivity analyses of the network model. We considered a reference scenario, which is defined by the instantiation of model parameters (initial state and rates) as specified in Table 1. When presenting and discussing our model results, we explored in some cases modelling options that lead to the definition of model parameters different from those in the reference scenarios.

The values of several kinetic parameters (i.e. those that are marked as “Fitted” in Table 1) were determined by systematically exploring the parameter space to find a satisfactory match with the experimental data. The dataset used for the fitting included measurements of one final output effector species (e.g., the *spollG* transcript), and one intermediate species in the network (KinA). Experimental data for the dynamics of these species under IPTG stimulation were obtained from [16]. The returned predictions of all the other experimental data used for evaluating the quality of the model were not used for the fitting. It is fair to note, that a clear consensus does not exist in the literature for the various parameters for which we determined the nominal value through fitting. For example, for the hill function parameters for the Spo0A regulation of *spoll* operons transcription, different sets of values are provided, see for instance the two papers published from the same group [16] and [18]. These discrepancies in the published values of parameters are clearly indicative of areas for further experimental quantification of the kinetics of various biochemical processes.

### The model is valid for inducible systems

Since this model is based on previous studies on inducible systems, we first analysed its ability to reproduce the available experimental observations for that class of systems.

**Figure 3:**
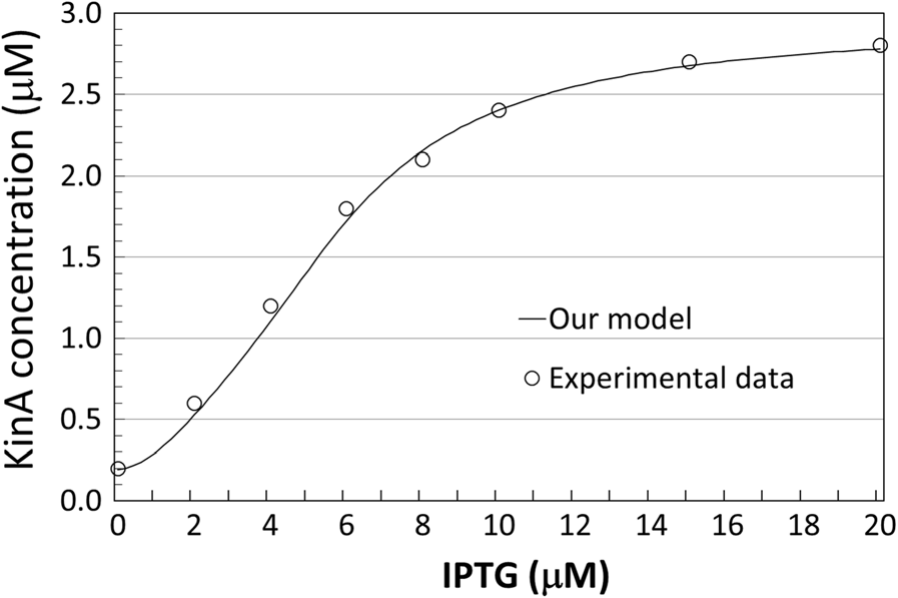
**Comparison of model prediction for KinA concentration upon varying levels of IPTG induction.** Total KinA concentration (on vertical axis, in μM), IPTG induction (horizontal axis, in μM): model predictions – continuous curve, experimental data (reported in Narula et al. [16]) – circles.

**Figure 4:**
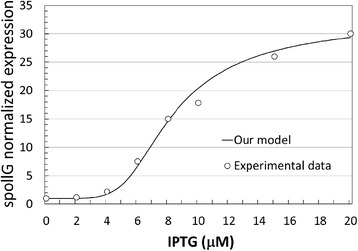
**Comparison of model predictions and experimental data for the normalized spollG expression levels.** Normalized levels of *spollG* expression are quantified through the concentration of the transcript and plotted on the vertical axis (in μM). IPTG concentration is on the horizontal axis (in μM). Model results are a continuous line experimental data plotted as circles. The level of expression is normalized with respect to the value when IPTG = 0 μM.

For KinA expression, we compared our model results with the experimental data reported in [16]. According to the quantitative experimental data reported in Figure S1C of the Supporting Information of [16], the total KinA concentration shows a variation in the range 200 μM-2800 μM when IPTG is increased from 0 μM to 20 μM – confirming that KinA expression level varies according to levels of the IPTG induction. Figure 3 shows our model’s prediction for the total KinA concentration compared to the published experimental data [16]. In the range of concentration between 4nM and 10nM, the model produces an increase in KinA concentration of about 100%, matching their experimental evidence. In addition, as it can be seen in the figure, the results predicted by our model are in good agreement with the experimental results published by Narula and colleagues [16]. To obtain a mathematical measure of the goodness of the agreement, we used a distance metric defined as follows: Given two sequences of *n* experimental data points {*o*_1_, *o*_2_, …, *o*_*n*_} and *n* model predicted data points {*m*_1_, *m*_2_, …, *m*_*n*_}, we take as the distance between the two data sets the following quantity:

$$ \frac{1}{n}{\displaystyle \sum_{i=1}^n}\frac{\left|{o}_i-{m}_i\right|}{o_i} $$

that is, we first compute the absolute distances between the experimental and model predicted data points, then divided each of those distances by the value of the experimental data, and finally we compute the average. For the data reported in Figure 4, the goodness of the agreement is equal to 4.03%.

Furthermore, our model is able to predict the concentration of the active Spo0A protein in the cell. For the inducible system being modelled, there are no direct experimental measurements of the active form of Spo0A available. Therefore, we used as an indirect reporter the levels of the Spo0A~P directly regulated *spollG* transcripts. We illustrate in Figure 4 the predictions returned by our model with the experimental data published by Narula and colleagues on the IPTG-dependent levels of expression of *spollG*. As presented in [16], the experimental concentration of *spollG* transcript was measured after 3 hours upon induction with an increasing level of IPTG (on the horizontal axis). The data in Figure 4 are normalized, dividing each value by the concentration when IPTG is zero. The goodness of the agreement is 8.84%.

**Figure 5:**
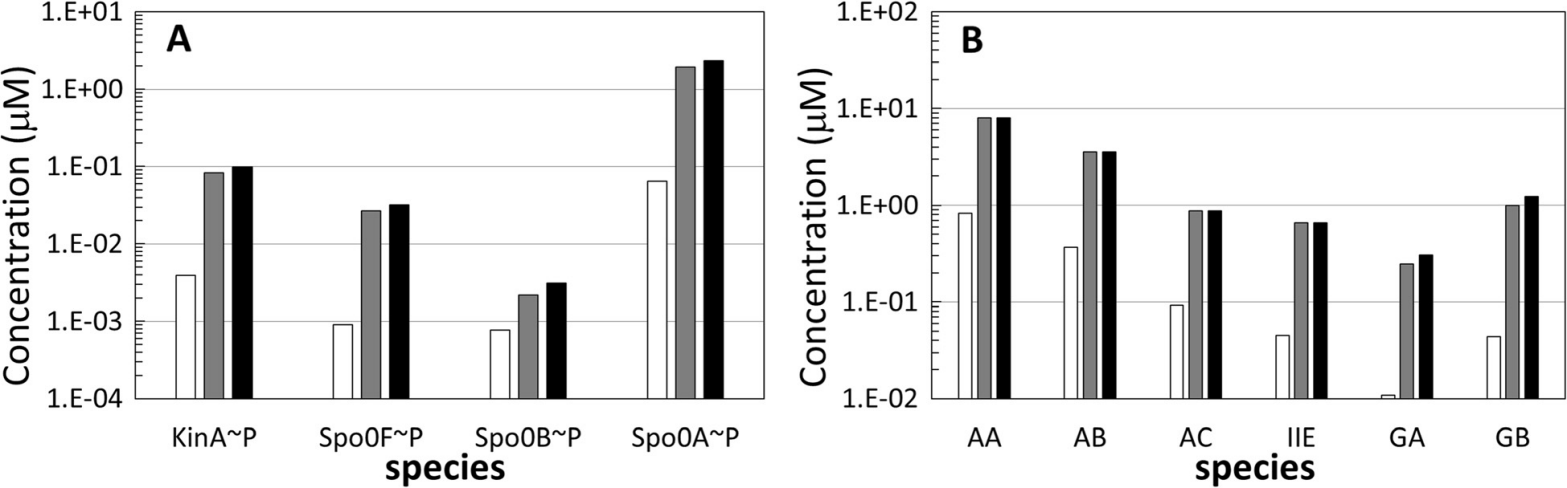
**Steady state concentration of model species for varying levels of IPTG induction. A**: Phosphorylated forms of phosphorelay proteins, in μΜ, when IPTG = 0 μM white bars, IPTG = 10 μM grey bars, IPTG = 20 μM black bars. **B**: Output proteins, in μM, when IPTG = 0 μM white bars, IPTG = 10 μM grey bars, IPTG = 20 μM black bars.

Finally, we show in Figure 5 a comparison between the steady state values of the concentrations of the phosphorelay species (left chart, A) and the effector proteins of the models, i.e. AA, AB, AC,IIE, GA and GB proteins (right chart, B), when IPTG is 0 μM (white bars), 10 μM (grey bars) and 20 μM (black bars). Notice that in the reference model parameters listed in Table 1, the value of the SS stimulus (representing the sporulation signal) is not zero, for which reason a basal level of phosphorylated KinA is observed even when the IPTG signal is absent. This basal level of phosphorylated KinA is sufficient to cause a weak activation of the phosphorelay, as shown by the white bars in Figure 5A. This weak activation leads to the transcription of genes regulated by spoll promoters and to the generation of low steady state concentrations of output proteins, as shown in Figure 5B by white bars.

This steady-state is representative of a situation where the cell is sensing unfavourable conditions of the environment but has not yet committed to sporulation. When the IPTG stimulus is included, the levels of KinA are artificially increased and so the amount of phosphorylated KinA is increased, leading to a stronger activation of the phosphorelay, as shown by the grey and black bars in Figure 5A. Moreover, the positive feedback exerted by Spo0A~P on KinA, Spo0F and Spo0A genes transcription further shifts the cell response to sporulation commitment. As shown in Figure 5B, AA, AB, AC and IIE proteins achieve their maximum levels when IPTG is equal to 10 μM, whereas GA and GB concentration levels show small additional growth when IPTG is increased from 10 μM to 20 μM. This difference in the dynamics is due to the different affinities that Spo0A~P has for the spoll promoters [24], which lead to the expression of GA and GB only at very high concentrations of Spo0A~P. These results are in line for the observed sporulation rate of IPTG stimulated cells.

### The Spo0A positive feedback on KinA is not essential for model output

We included in our model the positive feedback of Spo0A~P on KinA expression. From a point of view of the sporulation network, this is an important structural element, because it establishes a loop between the final effector output of the phosphorelay Spo0A and the originator species, i.e. KinA. However, the authors in [16] are able to obtain the same output behaviour in terms of the predicted Spo0A activation, without considering this feedback. So, we quantified the relevance of this feedback in the overall architecture of the model.

To check what the consequence of the exclusion of this link would be on the dynamics of the system, we ran a simulation where the contribution of Spo0A~ to KinA expression is set to null (parameter k_3_ of rate tr2 in Table 1). What we obtained is a response to IPTG of reduced intensity, but qualitatively very similar. Quite interestingly, the net effect of this reduction can be compensated by a very local change of the model. It is indeed sufficient to modify the values of the parameters that regulate KinA transcription through LacI repression (k_2_ and KL of rate tr2 in Table 1), without any other change in the model, to obtain levels of KinA expression that match the experimental ones.

**Figure 6:**
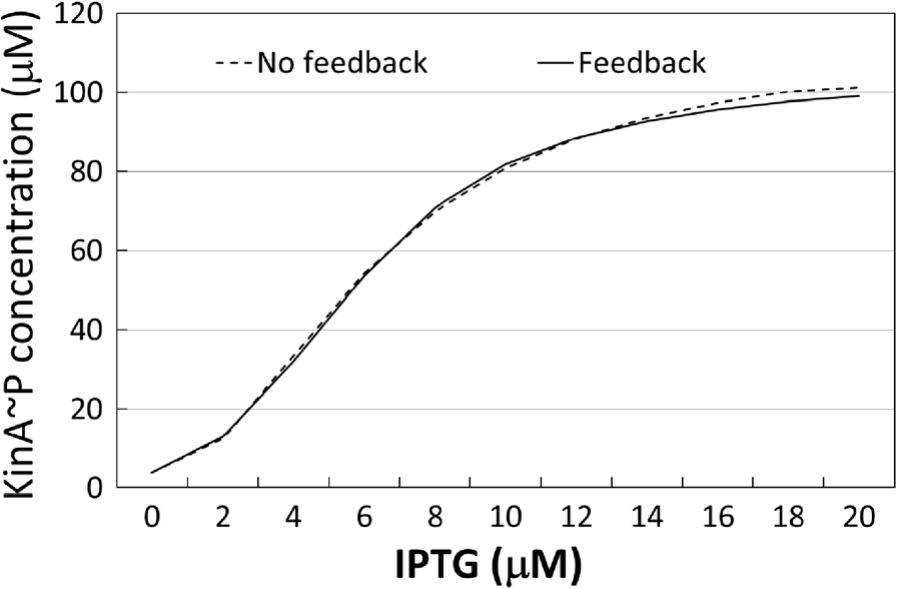
**Concentration of phosphorylated KinA for varying levels of IPTG induction.** Steady-state concentration of KinA ~ P, on the vertical axis, in μM, with varying level of IPTG induction strength, on the horizontal axis in μM, when the positive feedback loop of Spo0A~P on KinA transcription is removed (discontinuous curve) and when it is present (continuous curve).

We show in Figure 6 the steady-state level of phosphorylated KinA with varying levels of IPTG, when there is no feedback from Spo0A~P (dashed line) and when the feedback is applied (continuous line). As it can be observed, there is quite a negligible difference between the two curves, the goodness of the agreement being equal to 1.72%.

Since the levels of phosphorylated KinA are practically the same in the two modelling scenarios, it is expected that the level of the phosphorylated forms downstream in the phosphorelay will be the same. Therefore, the two modelling scenarios provide the same response.

### The phosphorelay motif transfers the dynamics of kinase activation onto the final effector Spo0A

**Figure 7:**
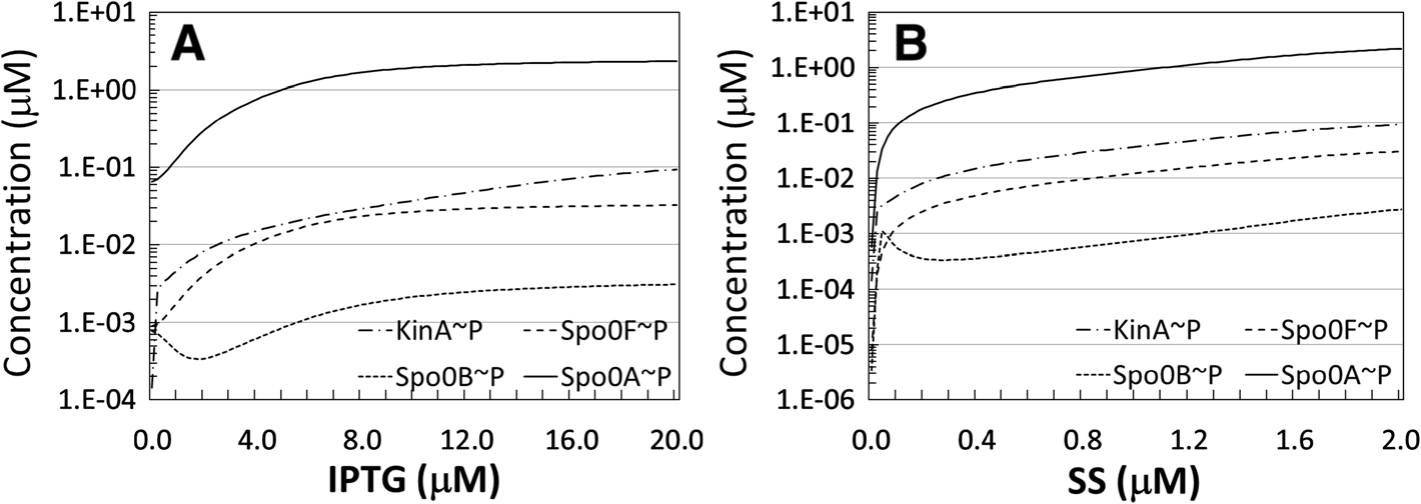
**Concentration of phosphorylated species for increasing levels of the sporulation signals. A**. Model predicted absolute concentrations, on the vertical axes, in μM, of the phosphorylated forms of phosphorelay proteins, with varying the strength of induction with IPTG, on the horizontal axis, in μM. **B**. Model predicted absolute concentrations, on the vertical axes, in μM, of the phosphorylated forms of phosphorelay proteins, when the IPTG stimulus is absent and the SS stimulus, on the horizontal axis, in μM, is applied.

The *B. subtilis* phosphorelay plays a central role in determining how the stimuli that trigger sporulation are relayed to the effectors. We explored further how the abundances of the phosphorylated forms of phosphorelay proteins are affected by those stimuli, looking at the predictions of their steady-state concentrations. Figure 7 shows the steady state concentrations of the phosphorylated species when IPTG is varied from 0 to 20 μM and SS is kept fixed to the nominal value in Table 1 (A) and when the IPTG stimulus is absent and SS is varied from 0 to 2 μM (B).

In both cases, all the species follow the same increasing trend, apart from Spo0B ~ P which is not monotonic. Notice that the vertical scale is logarithmic, and that in both cases (A and B) the stimulus is increased in a linear way. In Figure 7A, we can postulate that the sigmoidal response of the system is determined by the release of the cooperative inhibition on KinA transcription, whereas in Figure 7B the logarithmic shape of the three increasing curves is indicating that the response is linear. In both cases, the shapes of the curves for the first phosphorelay active species KinA ~ P is relayed in the phosphorelay down to effector species Spo0A~P.

### The model cannot reproduce both wild type and inducible systems

As shown in Figure 7, our model allows for activated sporulation through the increase of the SS signal. With a proper modulation of the SS signal, this form of induction should allow reproduction of the activation of the master regulatory protein Spo0A in the wild type.

**Figure 8:**
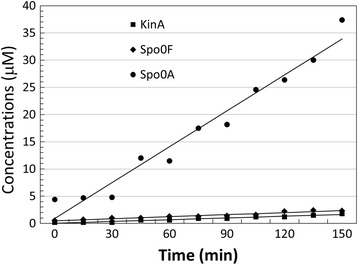
**Experimental measurements of phosphorelay species abundance in wild-type.** The experimental time-course data (units in minutes, on the horizontal axis) provided in [56] for the total concentration (phosphorylated and non-phosphorylated) of KinA, Spo0F and Spo0A species. The continuous lines are the best linear approximations, that we computed by linear regression using the R statistics package [62].

The experimental results, based on single-cell measurements, reported by Eswaramoorthy and colleagues in [56] indicated a linear growth of the total concentrations of each phosphorelay species upon sporulation initiation in *B. subtilis* wild-type (strain PYt9). We show in Figure 8 the experimental time-course data (units in minutes, on the horizontal axis) provided in [56] for the total concentration (phosphorylated and non-phosphorylated) of KinA, Spo0F and Spo0A species. The continuous lines are the best linear approximations, that we computed by linear regression using the R statistics package [62]. The linear approximations are qualitatively good, with R squared statistics 0.95, 0.93 and 0.95 for KinA, Spo0F and Spo0A, respectively.

The experimental data set does not show a sigmoidal shape. Although the data is for a time course and not for equilibrium, we would expect the positive feedback acting on KinA, Spo0F and Spo0A, to result in sharp increase of concentrations at the sporulation onset as opposed to linear trends. On the contrary, we find in this experimental data for wild-type a confirmation of the need for a gradual accumulation of the master regulatory protein Spo0A for a proper activation of the downstream targets, which has been suggested by several studies, for instance [18] and [20]. The amplitude of this linear increase in the total amount of species cannot be reproduced with our model. In particular, the total concentration of Spo0A cannot exceed 4.5 μM, while the experimental data from [56] that we report in Figure 8 show accumulation of total Spo0A up to 37.5 μM. This important discrepancy is indicative of a profoundly different mechanism of sporulation initiation and highlights important conflicts in the modelling studies our work is based on.

### The activation of the final effector Spo0A is not sensitive to Spo0B regulation

We note furthermore, that the experimental data reported by Eswaramoorthy and colleagues in [56] show a variation of Spo0B concentration as the sporulation commitment of the cell is enforced. More precisely, the experimental evidence shows the total amount of Spo0B has a linear increase as the sporulation process progresses.

This experimental evidence is in contrast to the consensus in literature that says that Spo0B is not regulated. The only work that reports a regulation of Spo0B is a recent paper by Carabetta *et al.* [63], which states that the phosphotransfer activity of Spo0B may be modulated by a ternary complex formed by YlbF, YmcA and YaaT proteins. The role of this complex appears to be the one of accelerating the phosphorylation of Spo0A, probably by interacting with Spo0F and Spo0B. This hypothesized mechanism of action cannot however explain the increase of the total amount of Spo0B demonstrated in [56].

**Figure 9:**
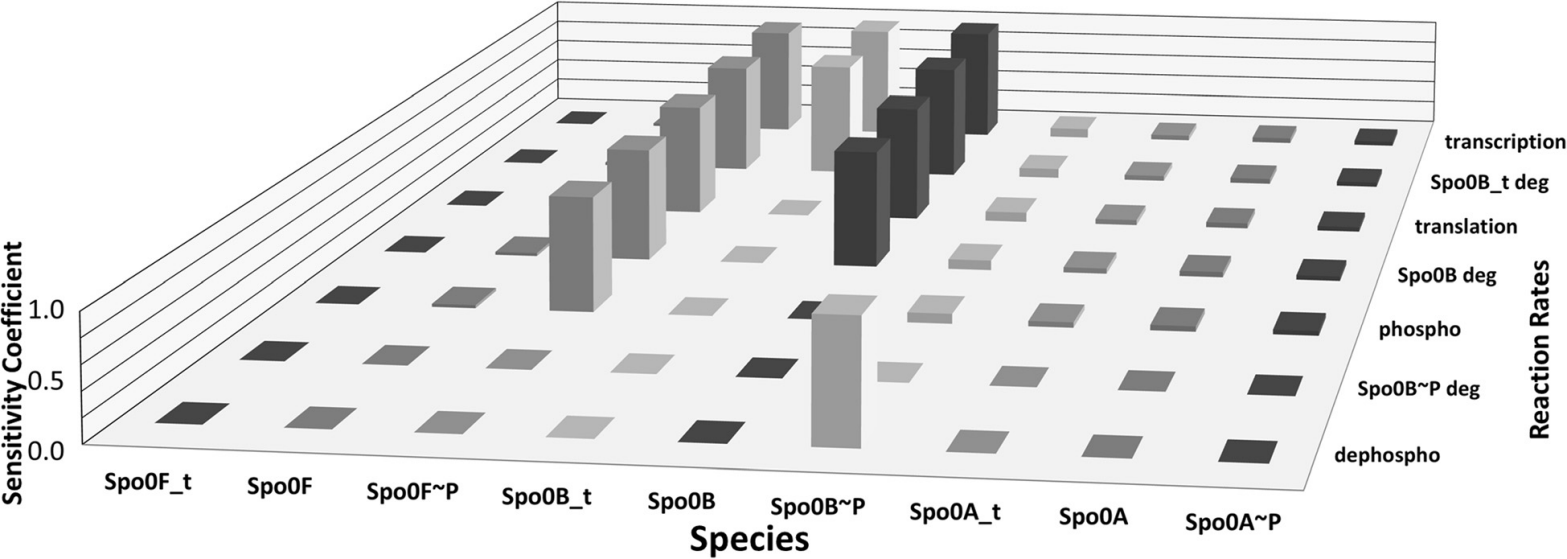
**Sensitivities of phosphorelay species to variations in the rates of Spo0B reactions.** Phosphorelay species on the horizontal front axis (transcripts, protein and phospho-forms), reaction rates on the horizontal lateral axis, sensitivity coefficients on the vertical axis.

We conducted parametric sensitivity analysis to elucidate what would be the dependence of the system on the regulation of Spo0B. We first used the local sensitivity coefficients of parameters computed by COPASI around the steady-state concentration values. Quite interestingly, the most sensitive are the phosphorelay species, and among the phosphorelay species, Spo0B ~ P is the species that shows the highest sensitivity coefficients. At the same time, we find that the changes in the reaction rates that affect the amounts of Spo0B species (transcript, protein and phosphor-form) have the least impact on the steady-state values. Figure 9 shows the sensitivity coefficients for the phosphorelay species Spo0F, Spo0B and Spo0A when the rates of Spo0B reactions are perturbed – we use a colour-scheme that assigns a grey scale proportional to the absolute value of coefficient. Obviously, changes in the reaction rate for Spo0B transcription, mRNA degradation, translation, protein degradation, phosphorylation and dephosphorylation (phospho-transfer to Spo0A) affect the steady-state concentration of the different Spo0B forms (darkest grey boxes, absolute value of the sensitivity coefficients greater or equal to 1). However, for the other phosphorelay proteins there is virtually no impact (lighter grey boxes, absolute value of the sensitivity coefficients between 0.05 and 0.1, lightest grey for values between 0.01 and 0.04), except for Spo0F~P, whose concentration is directly affected by the amount of Spo0B~P. It is worthwhile noting that the maximum value of all the sensitivity coefficients is around 1.0, which means proportionality in the change. This maximum value of sensitivity is achieved for the rate of Spo0B free dephosphorylation, yet the concentration of all the other species of the phosphorelay is not at all affected by changes in the dynamics of the reaction. In this sense, the phosphorelay is clearly exhibiting robustness against fluctuations of its kinetic parameter values.

**Figure 10:**
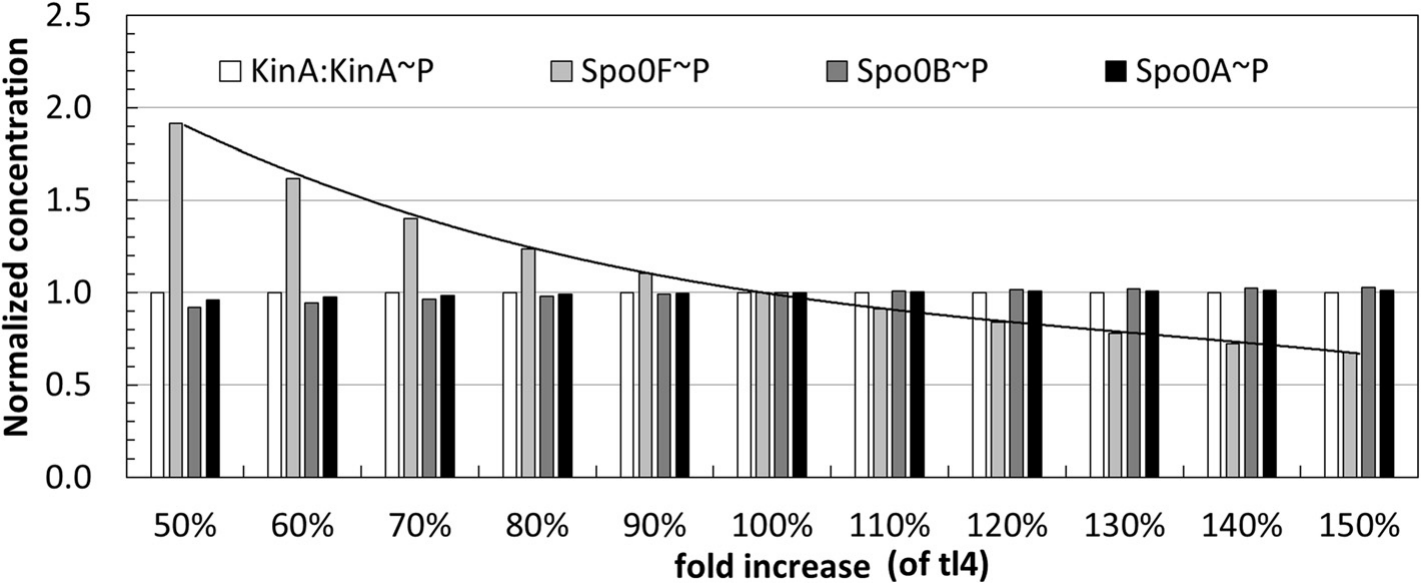
**Sensitivity of phosphorelay active forms of species with varying Spo0B translation rate.** Relative amounts of the phosphorylated species KinA:KinA, Spo0F, Spo0B and Spo0A, when the value of Spo0B translation rate constant varies in the interval [0.5-1.5] times its nominal value. All concentrations are normalized with respect to the value obtained when the translation rate takes the nominal value (central data point, when the fold increase is 100%).

To check whether this insensitivity is a local property that is valid only for the specific point of the parameter space defined by the model’s steady state, or if it is a more general property of the dynamics, we performed a global sensitivity analysis with respect to the parameters of synthesis, degradation and phosphorylation reactions that affect Spo0B total concentration. In this case, we varied the parameters in large intervals and we examined the corresponding changes on the phosphorelay species concentration. Figure 10 illustrates the result of one parameter scan performed when the rate constant value of the Spo0B translation reaction tl4 is varied in a range between 50% and 150% of its nominal value as described in Table 1 (variation corresponds to the interval [0.0538,0.2152]). In response to the perturbation of the translation rate there is a proportional increase of the total Spo0B concentration. As presented in Figure 10 the only species that is sensitive to the changes in the total amount of Spo0B is Spo0F~P (we added the trend line to make the trend clearer), whereas other species, including Spo0B~P, exhibit very little or no change.

This insensitivity poses an interesting question, which is, based on the experimental data reported by Eswaramoorthy et al. [56], why should the cell expend energy to increase the abundance of Spo0B during sporulation? A conjecture could be that Spo0B abundance increase, which is in accord to Figure 10, does not significantly affect the average value of Spo0A~P but may affect its variability, which we intend to examine by additional running stochastic simulation of sporulation models in future work.

## Discussion

In this paper we distilled the current molecular modelling knowledge about the sporulation initiation process in *B. subtilis*. In line with previous studies we built a model that includes an artificial sporulation initiation mechanism based on IPTG induction, to allow for tuneable regulation of the sporulation signal, and we also included a mechanism to model induction occurring in *B. subtilis* wild type. We included in the model, the positive feedback exerted by Spo0A~P on KinA, Spo0F and Spo0A transcription.

Our model predictions are validated against experimental data reported in several studies [14,16,56]. We found that it is difficult to reconcile the predicted dynamics determined by a model with the IPTG stimulus with those observed in the wild type and reported by Eswaramoorthy et al. in [56]. Indeed, while the model fits the experimental data for inducible systems, it cannot predict the amounts of phosphorelay proteins that are observed experimentally in wild-type *B. subtilis*.

This important discrepancy indicates that the reaction kinetics used in our model, which have been extracted from the literature, may not provide a faithful representation of the molecular mechanism for the wild type behaviour. To the best of our knowledge, there is a complete lack of experimental data describing the concentration of phosphorylated forms of the phosphorelay species. Under these circumstances it is impossible to determine what would be a more adequate modelling choice to elucidate the details of the phosphorelay dynamics. From our modelling results we can infer that some of the phosphorylated species, in particular Spo0F~P and Spo0B~P, would not be easily detected by experimental means as the phosphorelay dynamics reveals these to be parsimonious in their accumulation (see Figure 7). However for Spo0A~P our model predicts much higher concentration levels of the order of μM. The experimental data provided by Fujita et al. [24] indirectly suggests that Spo0A~P concentration needs to increase substantially in the cell to activate the downstream genes, given the low affinity that the species has for some of the gene promoters. It would be useful to conduct experiments that determine the dynamics of phosphorylated Spo0A in the wild type.

Finally, we would like to draw attention to the experimental data in [56], which shows that the total concentration of Spo0B, the central phosphorelay species, increases linearly over time during sporulation initiation. This highlights a gap in the knowledge of the biology of the phosphorelay as there is no evidence in the literature relating to Spo0B regulation. Moreover, we showed with sensitivity analyses (see Figure 10), that the steady-state response of the phosphorelay, including that of the main effector Spo0A~P, is quite insensitive to an increase of Spo0B concentration. This has led us to formulate an intriguing question concerning the increase in cellular levels of Spo0B which should have little effect on the downstream gene activation. To find possible answers we plan to explore stochastic simulations to ascertain whether variations in Spo0B concentration have an effect on the variability of the main effector Spo0A~P. The stochastic analysis of models of the phosphorelay of *B. subtilis* is a task that has been partially tackled by Csikász-Nagy et al. [64] and further investigations, may reveal behaviour that is additional to that established through continuous deterministic modelling.

## Conclusions

The modelling of the molecular network regulating sporulation in *B. subtilis* is a task that has so far been tackled by various studies, all based on IPTG inducible systems. In this paper we summarized the current published modelling understanding of sporulation initiation process into a model built with the COPASI modelling and simulation software tool. We found that while the model fitted the experimental data for inducible systems, it did not predict the amounts of phosphorelay proteins observed experimentally in wild-type *B. subtilis* [56]. This important discrepancy emphasized the necessity for additional experimental data. Specifically, we found no measurements for the abundance of the phosphorylated forms of phosphorelay proteins in wild-type *B. subtilis*, no knowledge available concerning the regulation of the phosphorelay protein Spo0B during sporulation, and variable measurements of reaction kinetics. In summary, we believe that the aforementioned issues may be some of the aspects currently impairing our ability in building mechanistic models at the molecular level of sporulation regulation in *B. subtilis* wild type.

## Additional files

Additional file 1:
**COPASI model file (application version 4.10, Build 55).**
Additional file 2:
**SBML model file.**
